# An Umbrella Review of Systematic Reviews and Meta-Analyses on Occupational Heat Exposure, Health Risks, and Productivity Losses Globally

**DOI:** 10.1007/s40572-025-00520-8

**Published:** 2026-01-07

**Authors:** Aditya Nath, Subhashis Sahu, Jason Kai Wei Lee

**Affiliations:** 1 Ergonomics & Occupational Physiology Laboratory, Department of Physiology, University of Kalyani, Kalyani, Nadia, West Bengal India; 2 Human Potential Translational Research Programme, Yong Loo Lin School of Medicine, National University of Singapore, Singapore, Singapore; 3 Department of Physiology, Yong Loo Lin School of Medicine, National University of Singapore, Singapore, Singapore; 4 Heat Resilience and Performance Centre, Yong Loo Lin School of Medicine, National University of Singapore, Singapore, Singapore

**Keywords:** Physiological strain, Renal impairment, Sperm quality, Workplace injuries, HSP70

## Abstract

**Background:**

Workplace heat exposure, intensified by climate change, increasingly threatens workers’ health, safety, and productivity, especially in the agriculture, construction, and manufacturing sectors. However, current evidence is fragmented due to varied study designs, and the absence of an integrated, multidisciplinary synthesis.

**Objectives:**

This umbrella review synthesizes findings from current systematic reviews and meta-analyses to appraise the health and productivity outcomes of workplace heat exposure, assess evidence quality, and identify critical research and policy gaps.

**Methods:**

Fourteen systematic reviews and meta-analyses (published up to 31st March 2025) were included following predefined *(PECOS)* criteria. Methodological fidelity was analyzed using the *AMSTAR checklist*, and the strength of evidence was evaluated using the *GRADE approach*.

**Results:**

The fidelity of the included reviews was rated from moderate to high, while the robustness of evidence spanned from low to moderate due to study heterogeneity and observational designs. Consistent evidence links workplace heat exposure to higher risks of heat-related illness, reduced eGFR (AOR = 3.50, 95% CI: 1.30–9.40) resulting renal impairment, cognitive decline, and injuries (1% increase in risk per 1℃ rises in temperature). Emerging findings suggests heat-induced sub-cellular and molecular damage (i.e., increased 8-OHdG, HSP70), reduced sperm quality, indicating cellular dysfunction. Women and relocated workers face greater physiological strain. Productivity losses affect 30–60% of exposed workers, with prior estimates suggesting annual global economic losses of approximately $2.1 trillion.

**Conclusions:**

Workplace heat hazards significantly threaten global workforce health and economic resilience. Urgent, coordinated interventions, robust policy measures, and high-quality longitudinal research are required to alleviate these risks.

**Supplementary Information:**

The online version contains supplementary material available at 10.1007/s40572-025-00520-8.

## Introduction

Workplace heat exposure significantly increases the risk of heat strain, a physiological condition resulting from the body’s inability to maintain thermal equilibrium, balancing continuous heat production and heat dissipation to maintain a body core temperature around 37 °C [1–5]. This imbalance is influenced by environmental factors, physical exertion, clothing insulation, and individual characteristics such as age and sex [6]. Prolonged exposure to high-temperature work environments can cause a cumulative heat burden, making heat stress a serious yet often underrecognized occupational hazard. However, the thresholds for heat sensitivity vary between different communities of workforces [7–13]. This resulted in 10.9% and 8.1% increments in emergency departmental visits and hospitalizations of the HRIs-affected agriculture workers and construction workers, respectively [14]. Around half a million people lost their lives globally due to extreme heat exposure during 2000-19 periods [15]. Extended workplace heat exposure also impairs the mental health of workers as well as increases the risk of suicide [16, 17]. The health risks are expected to intensify further as environmental temperatures continue to rise due to ongoing anthropogenic climate change. Global surface temperatures are projected to rise by 1.0℃−3.5℃ by the end of the 21 st century, with an increase of 1.09℃ observed between 2011 and 2020 compared to 1850–1900 [18, 19]. By 2023, anthropogenic global warming had surpassed approximately 1.31 °C above pre-industrial levels, with estimates ranging from 1.1 °C to 1.7 °C [20]. Climate change facilitates heat waves more frequently as extreme weather events globally, which were prevalent in 2024 worldwide [21]. Extreme heat waves disproportionately affect tropical and subtropical locales, though their global reach is expanding. In these areas, 30%−40% of annual day hours are not tolerable to sustain outdoor physical work [22, 23]. About 1.5% of global GDP will be lost by 2100 under the “RCP 6.0 (Representative concentration pathway 6.0)” and about 0.1% of global GDP will be decreased by the same under the “RCP 2.6 (Representative concentration pathway 2.6)” [24]. *Heat-related illnesses (HRIs)* are serious environmental and public health issues that need an epidemiological focus, and these should not be neglected [25, 26]. Workers across different sectors, such as agriculture, construction, mining, firefighting, and military service, are profoundly affected by workplace heat, often resulting in substantial productivity loss [27–29]. Such work hour loss ultimately intensifies the economic losses as well. Such outcomes have been increasingly observed across diverse global regions, underscoring the urgent need for prevention and mitigation strategies in different occupational settings [30, 31]. Our umbrella review aimed to systematically identify, synthesize, and evaluate corroboration from existing *systematic reviews and meta-analyses* on the health and productivity impacts of workplace heat exposure on workforces. It assesses the quality and strength of the evidence, identifies key research gaps, and outlines policy implications. This umbrella review integrates evidence across physiological, renal, cognitive, injury, molecular, and productivity domains, enabling a cross-domain comparison that reveals converging patterns, unresolved inconsistencies, and shared methodological gaps that no prior review has collectively examined.

## Methods

An identifiable, reproducible approach was utilized to ensure transparency and methodological completeness. Such a consistent approach ensured that high standards were consistently maintained throughout the entire process.

### Eligibility Criteria

Inclusion and exclusion benchmarks were developed using the *Population*,* Exposure*,* Comparison*,* Outcome*,* and Study Design* (*PECOS*) strategy [32].

#### Population

Workers exposed to workplace heat during work.

#### Exposure

Workplace heat exposure, extreme heat, or climate change-induced heat in the workplace.

#### Comparison

No comparison group was required, as the primary aim was to synthesize relationships rather than to directly compare the magnitude of effects.

#### Outcomes

Such outcomes include health outcomes (e.g., heat illness, injuries, reproductive health, and kidney function), physiological and cognitive effects, and productivity losses.

#### Study Design

Only systematic reviews and meta-analyses published in peer-reviewed journals were eligible for this umbrella review.

Excluded studies were reviews other than systematic reviews and/or meta-analyses, and that focused on the entire population other than workers of the type of interest, i.e., the general public or athletes. Reviews of animal studies were also excluded, in addition to any reviews for which full-text retrieval was unavailable.

*PubMed*,* Google Scholar*,* and Scopus databases* were utilized for an electronic search for the relevant systematic reviews and meta-analyses. Searches included articles published up to 31 st March 2025. The advanced search approach utilizing a blend of controlled vocabulary and free-text terms, such as: *(“heat stress” OR “heat exposure” OR “high temperature”) AND (“occupational” OR “workers” OR “labourers”) AND (“physical health” OR “mental health”) AND (“productivity” OR “economic outcome”)*. After that, filtering out the results to find the *“systematic review” AND “meta-analysis”.*

### Data Extraction and Management

Data were autonomously elicited by two reviewers, AN and SS. Extracted data included: author(s), year of publication, number of systematic reviews and meta-analyses included, population characteristics, occupational context, nature of heat exposure, primary outcomes, and key findings. Discrepancies were clarified through discussion or by involving a third reviewer, JKWL.

### Quality Assessment

Scientific fidelity of the incorporated systematic reviews was assessed using the *AMSTAR checklist *[33, 34]. In brief, the *AMSTAR checklist* consists of 11 elements (shown in Supplemental Material 1), each mandating evaluators to select from four possible responses: *Yes*, *No*, *Can’t answer*, or *Not applicable*. A numerical value of *1* is attributed to each *Yes* response, whereas all alternatives are accorded a value of *0*. Under guidelines established by the *Canadian Agency for Drugs and Technologies in Health*, *systematic reviews* are categorized by their cumulative scores as follows: *low quality* (0–3 points), *moderate quality* (4–7 points), and *high quality* (8–11 points) [35].

### Quality of Evidence

The *GRADE* (*Grading of Recommendations*,* Assessment*,* Development and Evaluations*) approach was employed to appraise the strength of evidence for key outcomes, grouping as *high*,* moderate*,* low*,* or very low* based on predefined criteria [36].

### Synthesis of Results

Due to the heterogeneity of the included reviews regarding outcomes, study populations, and exposure metrics, a narrative synthesis approach was adopted. Key findings were thematically grouped under: (i) health outcomes, (ii) occupational injuries, and (iii) productivity impacts.

## Results

### Study Selection

**Fig. 1:**
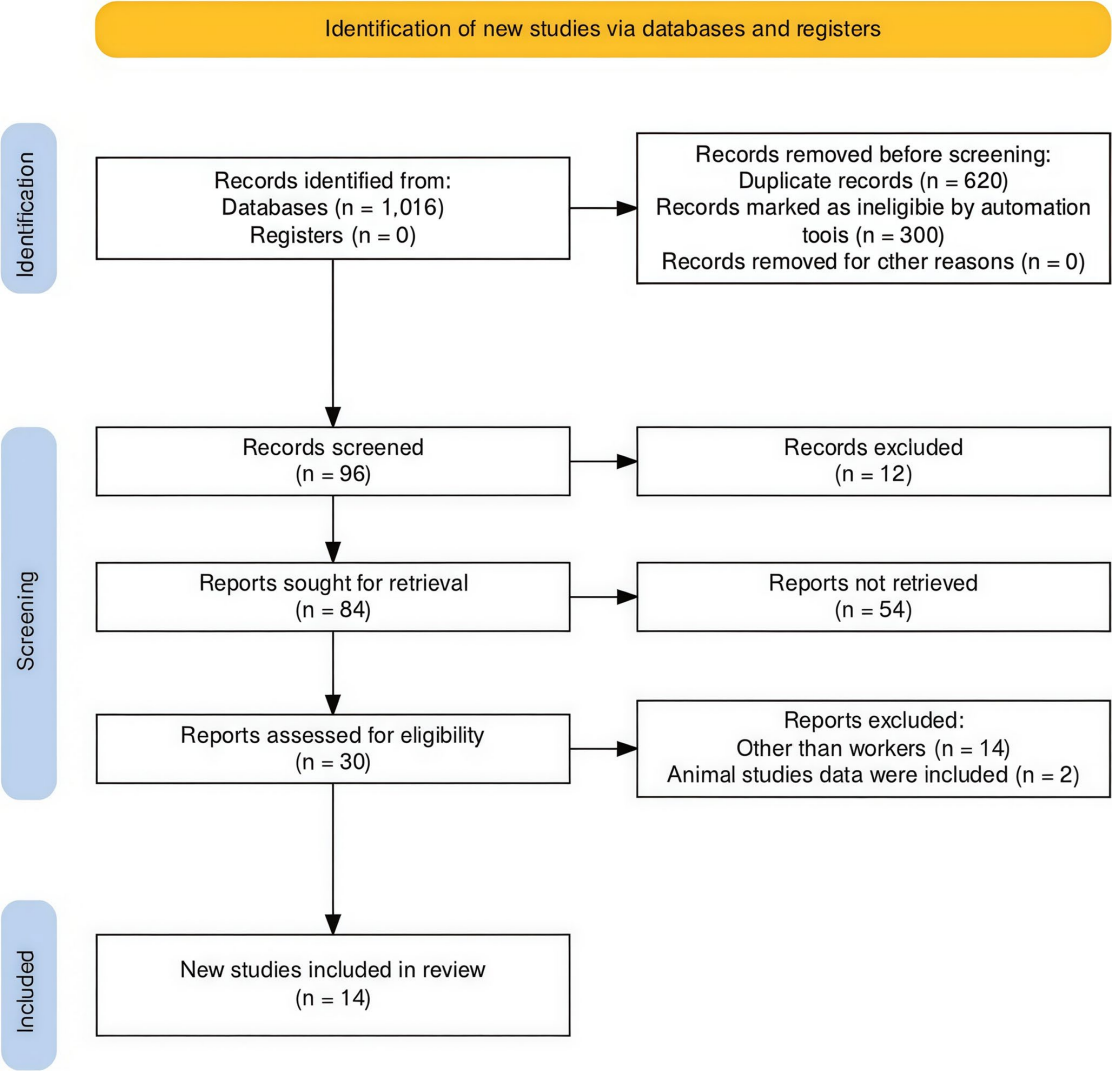
PRISMA Flow Diagram: study selection process for the umbrella review

The preliminary database search yielded a total of 1016 documents. After eliminating 620 duplicates, 300 records were removed after filtering the results for the *systematic review and meta-analysis*. 96 records were checked based on title and abstract. Of these, 30 full-text articles were assessed for suitability, leading to the final inclusion of 14 *systematic reviews and meta-analyses* in this umbrella review. The review selection process is outlined in Fig. 1 (*PRISMA* flow diagram) [37]. To assess the consistency of review (from 30 full-text systematic reviews and/or meta-analyses) inclusion between reviewers, Cohen’s kappa statistic was calculated [38]. The resulting value was κ = 0.863, indicating a strong level of inter-rater reliability. Discrepancies (*n* = 2) were resolved through discussion, along with the third reviewer. The details of Cohen’s kappa calculation were shown in Supplemental Material 2.

### Characteristics of Included Studies

The 14 included *systematic reviews and/or meta-analyses* were published between 2018 and 2024 and focused on a range of outcomes related to occupational heat exposure, including heat strain, injury risk, productivity loss, economic loss, kidney disease, and molecular biomarkers of DNA damage due to heat exposure. A majority of reviews targeted outdoor workers, such as agricultural and construction workers, with several considering climate change-induced heat stress.

### Quality of the Included Systematic Reviews and Meta-Analyses (*AMSTAR*)

**Fig. 2:**
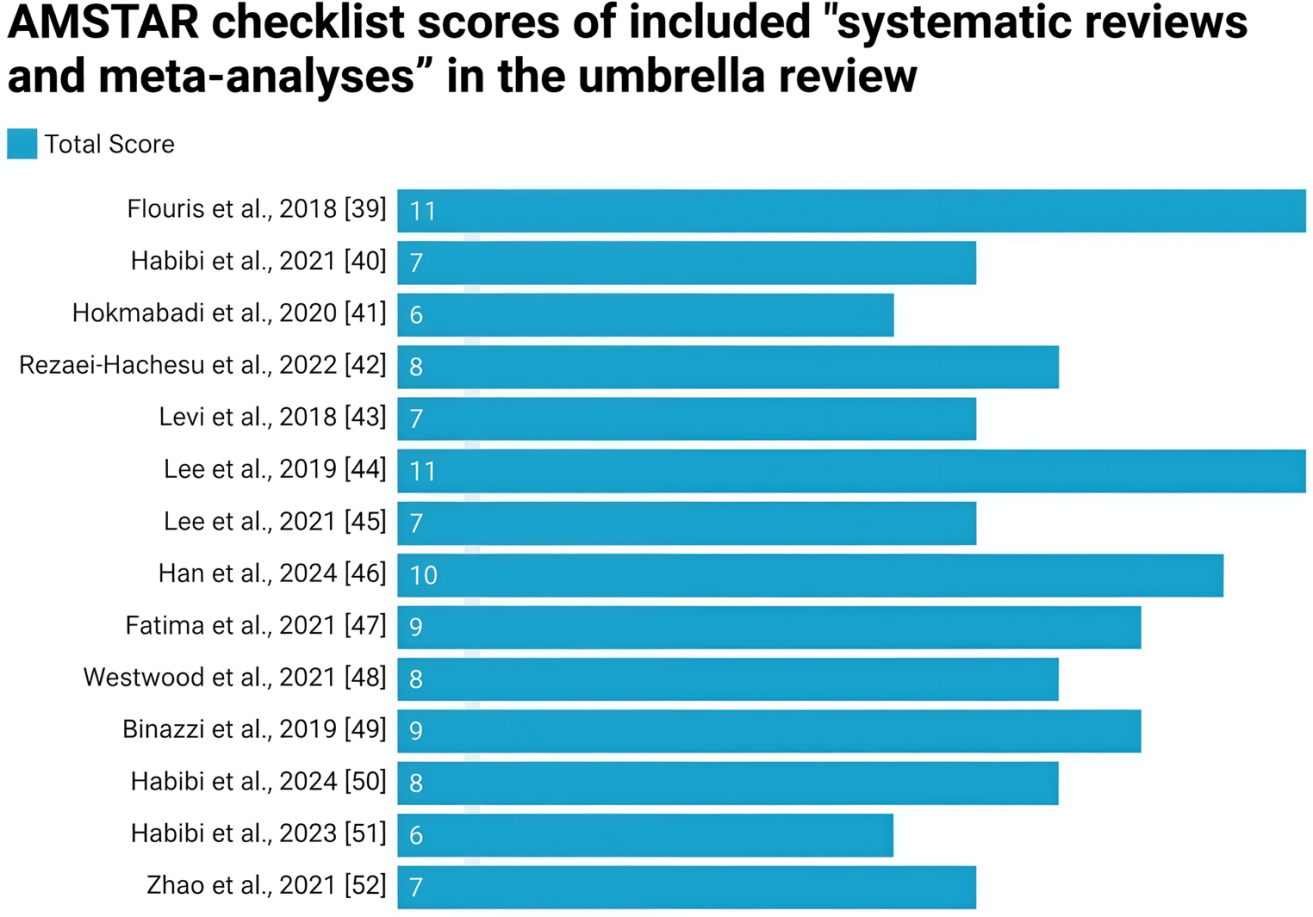
Methodological quality scores of the included systematic reviews and meta-analyses as assessed using the *AMSTAR checklist*

The overall quality of the included reviews was assessed using the *AMSTAR checklist* and is presented in Table 1. Among the 14 reviews, 8 were rated as *High* quality, and 6 were rated as *Moderate* quality. Total scores of the included reviews are depicted in Fig. 2.

**Table 1:** Highlights the quality of “systematic reviews and meta-analyses” included in the umbrella review

Serial No.	“Author(s) name(s) with year of publication”	“Q1”	“Q2”	“Q3”	“Q4”	“Q5”	“Q6”	“Q7”	“Q8”	“Q9”	“Q10”	“Q11”
1	Flouris et al., 2018 [39]	***“Y”***	***“Y”***	***“Y”***	***“Y”***	***“Y”***	***“Y”***	***“Y”***	***“Y”***	***“Y”***	***“Y”***	***“Y”***
2	Habibi et al., 2021 [40]	***“Y”***	***“Y”***	***“Y”***	***“CA”***	***“Y”***	***“Y”***	***“CA”***	***“Y”***	***“N”***	***“N”***	***“Y”***
3	Hokmabadi et al., 2020 [41]	***“Y”***	***“N”***	***“Y”***	***“N”***	***“Y”***	***“Y”***	***“Y”***	***“Y”***	***“N”***	***“N”***	***“CA”***
4	Rezaei-Hachesu et al., 2022 [42]	***“Y”***	***“Y”***	***“Y”***	***“Y”***	***“Y”***	***“Y”***	***“Y”***	***“N”***	***“CA”***	***“N”***	***“Y”***
5	Levi et al., 2018 [43]	***“Y”***	***“N”***	***“Y”***	***“N”***	***“Y”***	***“Y”***	***“Y”***	***“Y”***	***“N”***	***“N”***	***“Y”***
6	Lee et al., 2019 [44]	***“Y”***	***“Y”***	***“Y”***	***“Y”***	***“Y”***	***“Y”***	***“Y”***	***“Y”***	***“Y”***	***“Y”***	***“Y”***
7	Lee et al., 2021 [45]	***“Y”***	***“CA”***	***“Y”***	***“CA”***	***“Y”***	***“Y”***	***“Y”***	***“Y”***	***“N”***	***“N”***	***“Y”***
8	Han et al., 2024 [46]	***“Y”***	***“Y”***	***“Y”***	***“CA”***	***“Y”***	***“Y”***	***“Y”***	***“Y”***	***“Y”***	***“Y”***	***“Y”***
9	Fatima et al., 2021 [47]	***“Y”***	***“Y”***	***“Y”***	***“CA”***	***“Y”***	***“Y”***	***“Y”***	***“Y”***	***“Y”***	***“N”***	***“Y”***
10	Westwood et al., 2021 [48]	***“Y”***	***“Y”***	***“Y”***	***“CA”***	***“Y”***	***“Y”***	***“Y”***	***“Y”***	***“N”***	***“N”***	***“Y”***
11	Binazzi et al., 2019 [49]	***“Y”***	***“CA”***	***“Y”***	***“CA”***	***“Y”***	***“Y”***	***“Y”***	***“Y”***	***“Y”***	***“Y”***	***“Y”***
12	Habibi et al., 2024 [50]	***“Y”***	***“Y”***	***“Y”***	***“N”***	***“Y”***	***“Y”***	***“Y”***	***“Y”***	***“N”***	***“N”***	***“Y”***
13	Habibi et al., 2022 [51]	***“Y”***	***“Y”***	***“Y”***	***“N”***	***“Y”***	***“Y”***	***“CA”***	***“Y”***	***“N”***	***“N”***	***“CA”***
14	Zhao et al., 2021 [52]	***“Y”***	***“N”***	***“N”***	***“Y”***	***“Y”***	***“Y”***	***“Y”***	***“Y”***	***“N”***	***“N”***	***“Y”***

“Y”=yes, “N”=no, “CA”= can’t answer, “NA”= not applicable; “Yes”= 1-point, “all other responses”= 0 point

### Effect of Occupational Heat Exposure on Workers’ Health and Productivity

The outcome of workplace thermal exposure on labourers’ health and productivity is comprehensively summarized in Table 2, highlighting a consistent association between elevated heat stress and adverse health outcomes, as well as reduced labour productivity across diverse occupational settings.

**Table 2:** Presents the key findings, which have been thematically categorized into three primary domains such as: (i) adverse health outcomes, (ii) occupational injuries, and (iii) impacts on workers’ productivity

Serial No.	Author(s) name(s) with year of publication	Aim(s)	Number of studies	Number of participants/patients/cases	Study context	Type of analysis	Key findings
1	Flouris et al., 2018 [39]	Systematically reviewed and meta-analysed the impact of occupational heat strain on workers’ health and productivity outcomes.	111 studies for the systematic review, and from 111 studies, 64 studies were included for meta-analysis.	447 108 664 workers for systematic review and 55,791 workers for meta-analysis.	Workers’ health and productivity.	SRMA^a^	Meta-analyses revealed that workers engaging in one session under thermal stress conditions (wet-bulb globe temperature exceeding 22.0–24.8 °C, based on workload) demonstrated a 4.01-fold increased likelihood (“95% CI 2.45–6.58”; 9 scholarly works, 11,582 labourers) of experiencing occupational heat strain compared to those in thermoneutral environments. Furthermore, their internal body temperature elevated by 0.7 °C (0.4–1.0.4.0; 17 studies, 1,090 subjects), and *urine specific gravity* rose by 14.5% (0.0031, 0.0014–0.0048; 14 research articles, 691 participants). Among labourers completing shifts under heat stress conditions, 35% (31–39%; 33 studies, 13,088 workers) suffered from thermal strain, while 30% (21–39%; 11 research articles, 8,076 workers) reported diminished productivity. For individuals routinely exposed to heat stress (minimum 6 h daily, 5 days weekly, for at least 2 months annually), 15% (11–19%; 10 studies, 21,721 workers) developed renal impairment. The investigation incorporated diverse populations, exposures, and occupations to enhance the applicability of evidence synthesis, though this approach resulted in substantial heterogeneity across meta-analyses (71% to 99% of heterogeneity for six random effects of meta-analysis). *Grading of Recommendations*,* Assessment*,* Development and Evaluation* examination indicated moderate certainty for the majority of outcomes, with two measures (average internal temperature and changes in *urine specific gravity*) receiving very low confidence ratings due to industry funding of relevant studies.
2	Habibi et al., 2021 [40]	Systematically reviewed the impact of heat strain on outdoor workers.	25 studies included for systematic review.	Over 86,000 workers.	Outdoor workers’ health, productivity, and risk factors.	SR^b^	The existing evidence suggests a positive yet somewhat uncertain correlation between climate change-induced thermal strains in outfield-base workers. The utmost probable framework underlying this relationship includes both physiological and cognitive impairments, such as dehydration, fatigue, dizziness, delirium, diminished cognitive function, impaired concentration, and overall discomfort.
3	Hokmabadi et al., 2020 [41]	Systematically reviewed the consequences of workplace heat exposure, workplace injuries, and accidents.	30 research articles were included in the systematic review.	Over 46,000 workers, including both male and female workers.	Different workers from different occupational settings are frequently exposed to occupational heat.	SR^b^	The systematic review underscores the substantial effect of workplace thermal exposure on workplace safety, productivity, and the incidence of job-related injuries across various industries. Heat stress is vigorously associated with increased workplace accidents, including burns, slips, falls, and collisions, with heightened risks observed in sectors such as agriculture, construction, and manufacturing. Agricultural workers, particularly during peak summer months, face a significantly greater likelihood of sustaining traumatic injuries, with humidex values exceeding 33 correlating with a rise in accident rates. Likewise, employees in aluminium smelting industries are exposed to severe injury risks, with odds ratios reaching 2.28 and 3.52, respectively, as temperatures exceed critical thresholds (33–38 and above 38℃). Additionally, heat exposure has been shown to impair cognitive function, leading to slower reaction times and a greater frequency of errors in mentally demanding tasks, as evidenced by Stroop test results. Productivity declines notably in heat-affected industries, with rice and crop harvesters experiencing up to a 63.9% reduction in efficiency per hour due to prolonged exposure to high temperatures and direct sunlight. Moreover, excessive heat exposure has been linked to sunburn, fatigue, irritability, and sleep disturbances, all of which contribute to reduced work performance and a greater likelihood of occupational accidents. The incidence of labour compensation claims also increases by 6.2% under extreme heat conditions, highlighting the critical need for targeted intervention measures, such as hydration policies, shaded rest areas, flexible work schedules, and heat acclimatization programs, to mitigate the detrimental effects of occupational heat stress.
4	Rezaei-Hachesu et al., 2022 [42]	Systematically reviewed the effect of heat stress on renal function.	24 studies were included in the systematic review.	A total of 14,282 workers, including both male and female workers.	Male and female workers in exposed to occupational heat.	SR^b^	The findings from individual studies demonstrate considerable heterogeneity in populations, occupational settings, and renal function indicators, rendering a meta-analysis impractical. Multiple studies consistently reported that agricultural workers experienced a decline in *estimated glomerular filtration rate (eGFR)* and an increase in *serum creatinine (SCr)*, both indicative of kidney dysfunction. While interventions such as water, rest, and shade (WRS) programs showed some protective effects, kidney function continued to decline over work shifts. The prevalence of acute kidney injury (AKI) was notably high, with rates reaching 78% over three shifts, and heat stress (HS) was associated with a 1.29-fold increased likelihood of AKI (95%
							CI: 1.03, 1.61). Additionally, working in high-temperature environments significantly predicted reduced eGFR (OR = 3.50, 95% CI: 1.30, 9.40). Similar patterns were observed in metal industries, where heat-exposed workers had a significantly lower eGFR and an increased risk of renal impairment (OR = 2.9). Dehydration markers, including *urine specific gravity (USG)* and *blood urea nitrogen (BUN)*, consistently worsened from pre- to post-shift across industries such as mining, electric power, and construction. Among sugarcane workers, *USG* significantly increased across shifts, and heat exposure in various sectors was linked to higher odds of dehydration (OR = 3.6). However, some studies, particularly those focusing on steel industries and specific mining and electrical sectors, reported no statistically significant differences between heat-exposed and non-exposed workers. Overall, while substantial evidence indicates that *HS* contributes to renal dysfunction through dehydration and impaired kidney function, inconsistencies across studies underscore the need for further research to elucidate industry-specific risks and identify effective protective measures.
5	Levi et al., 2018 [43]	Systematically reviewed the consequences of occupational heat exposure on workers’ health and productivity outcomes.	154 research articles were incorporated in the systematic review.	Not mentioned.	Workers’ health and productivity.	SR^b^	Climate change has a significant influence on occupational health and productivity, particularly by intensifying the risks associated with workplace heat exposure. Rising global temperatures, driven by climate change, pose substantial challenges for workers engaged in physically demanding and outdoor occupations, including agriculture, construction, and manufacturing. Prolonged exposure to excessive heat heightens the likelihood of heat-related illnesses such as heat exhaustion, heat stroke, and chronic kidney disease, with the most severe effects observed among workers in hot and humid environments. Furthermore, heat stress contributes to dehydration, cardiovascular strain, and cognitive impairment, all of which negatively impact productivity, increase the risk of workplace accidents, and lead to long-term health consequences. The study highlights that low- and middle-income countries bear a disproportionate burden due to limited access to cooling interventions, insufficient occupational safety regulations, and broader socio-economic vulnerabilities. In addition to its health implications, heat stress results in economic losses by diminishing work capacity, slowing industrial productivity, and reducing earnings in labour-intensive sectors.
6	Lee et al., 2019 [44]	Systematically reviewed and meta-analysed the consequences of thermal strain on workers’ renal function and disease.	11 research articles were included in the systematic review and meta-analysis.	888,026 cases.	Workers’ kidney function.	SRMA^a^	The comprehensive assessment demonstrates that elevated temperatures correlate with a 30% elevation in kidney disease morbidity (95% CI: 20% to 40%). Analysis by disease classification revealed statistically significant correlations for both renal colic/kidney stones and additional renal pathologies. Furthermore, stratified analyses according to research methodology identified significant outcomes in time-series investigations and studies utilizing alternative methodological approaches. Regarding temperature measurement parameters, substantial effects were observed in studies employing mean temperature metrics, as well as those investigating heat wave phenomena or heat stress conditions. Throughout all investigations and subgroups, I² statistics ranged from 21.9% to 80.2%, indicating moderate statistical heterogeneity. Considering that I² values exceeded 50% across all analyses, the implementation of a random-effects model was determined to be methodologically appropriate.
7	Lee et al., 2021 [45]	Systematically reviewed the outcome of heat exposure on workers’ health.	47 scholarly articles were included in the systematic review.	151,421,924 workers.	Workers’ health status.	SR^b^	Extreme temperatures present serious health hazards for workers, with a growing body of evidence highlighting concerning trends across various occupational sectors and demographic groups. Men, ethnic minorities, and individuals engaged in labour-intensive occupations such as construction, agriculture, and mining are disproportionately affected by heat-related illnesses (HRIs). At the same time, young adults aged 15 to 35 and residents of rural areas also exhibit heightened vulnerability. Research indicates a marked increase in emergency department visits and hospitalizations as ambient temperatures rise, particularly in regions with a higher density of outdoor workers. Periods of consecutive hot days are associated with a 30% increase in all-cause mortality, with manual labourers at especially elevated risk. HRIs become significantly more prevalent at temperatures exceeding 35.5 °C, with incidence rates rising as much as sevenfold during heat waves. Field studies report commonly experienced symptoms such as profuse sweating, headaches, dizziness, and muscle cramps, with female workers nearly three times more likely than males to report multiple symptoms. Construction workers demonstrate near-universal symptom prevalence, while consistently high rates of HRI are also reported among farmworkers and miners. Occupational mortality linked to heat exposure is particularly pronounced among agricultural labourers and Hispanic workers, with elevated cardiovascular mortality observed in vulnerable populations such as Nepali migrant labourers and French potash miners. The incidence of occupational injuries also rises during heat waves and prolonged hot periods, with risk factors including worker age, gender, company size, and sector of employment. Beyond immediate symptoms, heat exposure has significant physiological consequences. The urinary system is notably affected, with increased urolithiasis rates among spray painters and sugarcane cutters, largely attributed to dehydration and kidney dysfunction. Reproductive health outcomes include reduced sperm quality, delayed conception, and higher risks of caesarean delivery. Psychological and cognitive impairments have also been associated with prolonged heat exposure, often due to stress hormone responses and dehydration. Other documented health effects include dyslipidaemia, cardiovascular strain, and eye problems, especially among outdoor workers such as rice harvesters. Taken together, these findings underscore the multifaceted and far-reaching health implications of occupational heat exposure, emphasizing the urgent need for targeted interventions to safeguard vulnerable worker populations across diverse industries and geographic regions.
8	Han et al., 2024 [46]	Meta-analyses the impact of occupational heat exposure on construction workers’ productivity.	14 studies were incorporated in the meta-analysis.	2387 workers.	Construction workers’ productivity.	MA^c^	The analysis demonstrates that 60% of construction workers (95% CI: 0.48–0.72, *p* < 0.01) experience substantial productivity declines when exposed to high temperatures, particularly when Wet Bulb Globe Temperature exceeds 28 °C or ambient temperatures surpass 35 °C, with older workers (over 38) showing heightened vulnerability (61% reporting decreased productivity; 95% CI: 0.49–0.72) and teams including female workers exhibiting 26% greater likelihood of reduced output (ratio = 0.74, 95% CI: 0.60–0.87); these findings highlight the urgent need to address occupational heat exposure in construction through policies prioritizing protection for vulnerable demographics, development of heat-resilient technologies and ergonomic tools, implementation of environmental modifications to improve working conditions, and investment in longitudinal research to better understand long-term health impacts and develop site-specific microclimate management strategies that safeguard both worker wellbeing and productivity.
9	Fatima et al., 2021 [47]	Systematically reviewed with meta-analyses the effect of extreme heat exposure on work-related injuries in different climatic regions.	24 studies were included for the systematic review, and from 24 studies 22 studies were included for the meta-analysis.	A total of 5,847,583 workers, along with the cases.	Different occupational workers’ injury risks due to exposure to extreme heat.	SRMA^a^	A meta-analysis encompassing nearly 22 million occupational injuries (OIs) from six nations, such as Australia, Canada, China, Italy, Spain, and the USA, revealed that heat exposure significantly increases injury risk. Specifically, for each 1 °C elevation in temperature above reference levels, the cumulative likelihood of “OI” enhanced by 1% (“RR = 1.010, 95% CI: 1.009–1.011”), and during extreme heat events, the risk rose by 17.4% (“RR = 1.174, 95% CI: 1.057–1.291”). Across climatic regions, the Humid Subtropical Climate exhibited the highest “OI” risk during elevated temperatures (“RR = 1.017, 95% CI: 1.014–1.020”), followed by Oceanic (“RR = 1.010”) and Hot Mediterranean climates (“RR = 1.009”). During HW events, Oceanic and Humid Subtropical Climates again showed the highest OI risks, with RRs of 1.218 and 1.213, respectively, though no studies addressed Tropical regions. The impact of heat on OI was acute across all climate zones, typically manifesting within a 1–2-day lag. Certain demographics and occupational sectors were disproportionately affected, particularly young workers under 35, males, and those engaged in agriculture, forestry, aquatic harvesting, construction, manufacturing, power supply, gas, and water sectors. These groups faced heightened injury risks both during hot days and heat wave conditions. Inter-study heterogeneity (I²) was generally substantial, with I² values ranging from 0.0% to 91.3% across various climate zones and an overall heterogeneity of 89.3%. The findings highlight that the relationship between extreme heat and OI is modulated by climatic context, individual worker characteristics, and occupational environment. Given these disparities, the study emphasizes the need for tailored, climate-specific interventions and further research to address heat-related occupational risks across diverse worker populations and geographic settings.
10	Westwood et al., 2021 [48]	Systematically reviewed the individual risk factors associated with developing exertional heat illnesses.	42 articles were included for the systematic review.	Not mentioned.	Workers from different occupational settings who are exposed to occupational heat.	SR^b^	A review of 42 studies identified a broad spectrum of risk factors associated with exertional heat illness (EHI) among military personnel, primarily clustered into four domains: lifestyle, health, work-related constraints, and miscellaneous factors. Lifestyle-related risks were the most frequently reported, with being overweight and having low physical fitness emerging as the most significant contributors. Additional but less frequently noted lifestyle factors included smoking, alcohol intake within 48 h, use of illicit drugs, and consumption of caffeine or protein supplements. Health-related risk factors, while less studied, highlighted a heightened vulnerability to EHI following mild systemic illnesses such as colds, gastrointestinal disturbances, or febrile episodes—largely due to dehydration. Notably, dehydration was a recurrent theme across studies, often exacerbated by underlying illness. Work-related constraints also played a key role in EHI susceptibility, with evidence indicating that sleep deprivation, poor heat acclimatization, inadequate nutrition, and lack of experience substantially increased risk. Importantly, most studies emphasized that these factors do not operate independently; rather, lifestyle, health, and occupational variables often intersect to heighten vulnerability. While 22 studies clearly illustrated this interplay, 28 studies also pointed to additional, unspecified contributors to EHI risk. Collectively, these findings underscore the multifactorial nature of EHI and emphasize the need for integrated prevention strategies that address personal, health-related, and occupational determinants of heat illness.
11	Binazzi et al., 2019 [49]	Meta-analyses the impact of occupational heat exposure on workplace injuries.	8 studies were included in the meta-analysis.	16,709,848 workers.	Different occupational workers’ workplace injury risks due to exposure to extreme heat.	MA^c^	The meta-analysis examining the relationship between temperature and occupational injuries synthesized data from multiple studies, each employing different temperature measurement methods. The analysis focused on the highest daily temperature or thermal index (e.g., WBGTmax or Humidex) recorded during each study period to standardize comparisons. Risk estimates were primarily expressed per 1 °C increase in maximum daily temperature (32–37 °C) or based on temperatures within the 95th–99th percentile thresholds (35.5–37.1 °C). The analysis also accounted for injury compensation claims during the hottest months, with data spanning from 1994 to 2014. Separate evaluations were conducted for time-series and case-crossover designs, followed by a pooled analysis excluding agriculture-specific studies. Heterogeneity testing revealed significant variability in most groups (*p* < 0.001), justifying the use of a random effects model, except in the case-crossover subgroup (*p* = 0.522), where a fixed-effects model was appropriate. The combined relative risk (RR) for occupational injuries was modest yet statistically significant: 1.002 (95% CI: 0.998–1.005) for time-series studies, 1.014 (95% CI: 1.012–1.017) for case-crossover studies, and 1.005 (95% CI: 1.001–1.009) overall. Subgroup analyses showed slightly elevated RRs for male workers, individuals under 25, and those in agriculture, although not all findings reached statistical significance. No clear association was found for indoor/outdoor work settings or the construction sector. The findings highlight subtle but consistent increases in occupational injury risk with rising temperatures, particularly among younger and agricultural workers, while reinforcing the need for nuanced methodological approaches in evaluating heat-related workplace risks.
12	Habibi et al., 2024 [50]	Systematically reviewed the outcome of thermal stress on the women’s workforce.	13 studies were included in the systematic review.	1,233,539 workers.	Women workers’ health risks due to occupational heat exposure.	SR^b^	Scientific evidence indicates a positive, albeit imprecisely defined, association between climate change-driven heat strains among female workforces. The impacts of occupational heat strain in this population include a range of physiological and cognitive issues such as fatigue, thermal discomfort, dehydration, diminished cognitive function, and impaired concentration. Climate change is projected to increase both the frequency and intensity of heat-related illnesses, as well as elevate the risk of work-related injuries. Moreover, the broader health consequences for women exposed to excessive occupational heat in changing climatic conditions are a growing concern. This systematic review highlights the urgent need for targeted research to deepen our understanding of how climate change exacerbates occupational heat strain specifically in women workers. There is robust evidence supporting the notion that ongoing climate change will continue to intensify these occupational hazards. Consequently, it is imperative to develop and implement preventive interventions grounded in multidisciplinary approaches. Such strategies should aim to minimize the health risks experienced by women workers in high-temperature environments and reduce the broader negative implications of climate change on their well-being and productivity.
13	Habibi et al., 2022 [51]	Systematically reviewed regarding the biomarkers of heat-induced DNA damage used for diagnosis.	7 studies were included in the systematic review.	2010 workers.	Different occupational workers’ DNA damage due to heat exposure.	SR^b^	The analysis revealed several diagnostic biomarkers indicative of heat stress-induced DNA damage due to occupational exposure. These include urinary 8-hydroxy-2′-deoxyguanosine (8-OHdG), micronuclei in peripheral blood lymphocytes, heat shock proteins (HSP70), testosterone levels, leukocyte counts, and assessments of sperm and semen quality. These biomarkers are effective in detecting sub-cellular and molecular damage caused by prolonged exposure to high-temperature work environments. The findings underscore the critical occupational health risks posed by thermal stress, which can lead to cellular and DNA-level damage in vulnerable worker populations. The study concludes that identifying and validating molecular biomarkers such as HSPs and 8-OHdG is essential for the early detection of heat-induced cellular injuries. Implementing diagnostic tests based on these markers can play a pivotal role in preventing heat-related health disorders among workers. Furthermore, the study highlights the urgent need for more research in tropical and subtropical regions to develop and validate novel biomarkers tailored to high heat exposure scenarios.
14	Zhao et al., 2021 [52]	Systematically reviewed the impact of heat-related productivity losses on the economy.	30 articles were incorporated in the systematic review.	Not applicable.	Heat-induced productivity losses are associated with the economic crisis.	SR^b^	This systematic review of existing literature examines the methodologies used to assess the economic consequences of heat-induced declines in labor productivity. Four main approaches emerge from the review: the “human capital” (HC) method, the “econometric method” (EM), the “input–output” (IO) method, and the “computable general equilibrium” (CGE) model. These models yield varying estimates of global economic losses, depending on assumptions about future climate scenarios and adaptation strategies. Under projected climate pathways, global GDP losses due to heat-related productivity declines by 2100 range from 0.31% (under RCP2.6) to 2.6% (under RCP8.5). The most significant losses are observed in regions with high heat exposure and labour-intensive economies, notably South and Southeast Asia, Sub-Saharan Africa, and Central America. However, methodological inconsistencies and differing considerations of adaptation efforts contribute to wide variations in outcomes—even up to a 7.4-fold difference within the same region and time frame. The review also identifies critical knowledge gaps in current research and calls for improved, standardized methodologies that account for localized conditions and evolving adaptation capacities. Addressing these gaps is essential to generate more accurate and actionable insights for shaping future climate and labor policies.

^a^SRMA= Systematic review and meta-analysis, ^b^SR= Systematic review and ^c^MA= Meta-analysis

### Quality of Evidence (*GRADE*)

Using the *GRADE approach*, the quality of evidence was evaluated for each major outcome across *systematic reviews and meta-analyses* highlighted in Table 3.

**Table 3:** *GRADE approach* applied to the included studies to find out the strength of evidence based on different outcomes reported in different systematic reviews and meta-analyses

Serial No.	Study	Outcome Focus	Certainty (*GRADE*)	Justification
1	Flouris et al., 2018 [39]	Occupational heat strain, productivity, and kidney outcomes.	Moderate	Strong SRMA^a^; downgraded for heterogeneity.
2	Habibi et al., 2021 [40]	Heat strain effects on outdoor workers’ health.	Low	Well-conducted review; some inconsistency and small-study limitations
3	Hokmabadi et al., 2020 [41]	Workplace injuries from heat exposure.	Low	Observational studies with limited control.
4	Rezaei-Hachesu et al., 2022 [42]	Heat exposure and kidney disorders.	Low	Limited studies and large methodological heterogeneity.
5	Levi et al., 2018 [43]	Climate-related heat stress and workers’ health and productivity.	Moderate	Narrative synthesis of evidence downgraded due to high heterogeneity.
6	Lee et al., 2019 [44]	High temperature and kidney morbidity.	Low	Meta-analysis with sound methodology, but downgraded for study variability and geographic limitations.
7	Lee et al., 2021[45]	Heat exposure and general worker health.	Low	Some inconsistency between occupational groups.
8	Han et al., 2024 [46]	Heat exposure and construction workers’ productivity.	Moderate	Targeted population; clear findings, but downgraded for limited generalizability.
9	Fatima et al., 2021 [47]	Extreme heat and occupational injuries.	Moderate	Cross-sectional evidence; downgraded for risk of bias and indirectness of population representation.
10	Westwood et al., 2021 [48]	Individual risk factors for heat illness.	Low	High inconsistency, limited number of robust trials, and indirectness in outcome relevance.
11	Binazzi et al., 2019 [49]	Heat stress and occupational injuries.	Moderate	Consistent findings; downgraded for observational nature and unadjusted confounding.
12	Habibi et al., 2024 [50]	Heat strain in women workers.	Low	Gender-specific review; downgraded for small sample sizes and variability in exposure metrics.
13	Habibi et al., 2022 [51]	Diagnosing biomarkers of DNA damage from heat.	Low	Cutting-edge biomarkers, but experimental designs with indirect clinical translation.
14	Zhao et al., 2021 [52]	Economic impact of heat on productivity.	Moderate	Clear link to labor output; downgraded due to variability in economic assessment methods.

^a^SRMA= Systematic reviews and meta-analysis

## Discussion

This umbrella review synthesizes the existing evidence on the health and economic consequences of occupational heat exposure, drawing from 14 systematic reviews and/or meta-analyses across diverse working populations, work settings, and climatic zones. In sum, these findings confirm that occupational heat stress represents a major public health and workforce concern, particularly for those engaged in outdoor or industrial work under high-temperature conditions. The included systematic reviews and meta-analyses are consistently supportive of broad associations between elevated thermal exposure, increased physiological strain, impaired health, and declines in work performance and productivity. Eight of the reviews were rated as being of high quality and six as being of moderate quality. The *GRADE* certainty of evidence is generally low to moderate because of heterogeneity and reliance on observational studies. Exposure metrics also differed among the reviews, with some using WBGT, others UTCI or humidex, and ambient air temperatures, thus again limiting comparability. While most syntheses pointed in a similar direction, heterogeneity in context and methodology indicates that these findings should be regarded as no more than suggestive. Overall, the current body of review-level evidence is supportive of the likelihood that prolonged exposure to heat contributes to physiological strain and reduced work output in affected populations.

### Health Impacts of Occupational Heat Exposure

Six reviews provide good evidence that occupational heat exposure is related to increased physiological strain and adverse health outcomes. Specifically, Flouris et al. [39] gave meta-analytic evidence that occupational heat strain increases fourfold in workers exposed to WBGT levels above 22.0–24.8 °C, depending on work intensity, alongside a 0.7 °C rise in body core temperature. Habibi et al. [40], Levi et al. [43], and Lee et al. [45] reported similar patterns among outdoor workers. They found that the likelihood of experiencing heat-related illness symptoms and thermal strain increased markedly when ambient temperatures rose above 35.5 °C during work. Impairment of sperm quality and an increase in the risk of infertility have also been associated with workplace heat exposure [45]. Rezaei-Hachesu et al. [42] and Lee et al. [44] examined renal function and reported that higher ambient temperatures were associated with reduced estimated glomerular filtration rate (eGFR; OR = 3.50, 95% CI 1.30–9.40), a 3.6-fold increase in dehydration risk, and a 30% higher likelihood of kidney disease. Although these two reviews analyzed different primary studies, they point toward a possible renal pathway underlying physiological burden due to heat exposure. Overall, the evidence suggests that heat exposure may contribute to physiological strain but does not establish a direct causal pathway.

### Occupational Injuries and Cognitive Impairment

Three systematic reviews and/or meta-analyses, such as Hokmabadi et al. [41], Fatima et al. [47], and Binazzi et al. [49], identified a clear link between rising temperatures and increased occupational injury risk. Hokmabadi et al. [41] reported significantly higher rates of traumatic injuries among agricultural workers during peak summer months, particularly when humidex values exceeded 33, and noted substantially elevated injury risks among aluminium smelting workers, with odds ratios of 2.28 and 3.52 at temperatures above 33 °C and 38 °C, respectively. Fatima et al. [47] supported these findings, observing a general trend of increasing injury rates, typically around 1% per 1 °C rise, though effect sizes varied by sector and climate. Binazzi et al. [49] further confirmed this association through pooled estimates, showing modest but statistically significant increases in injury risk, with relative risks of 1.005 (95% CI: 1.001–1.009). Westwood et al. [48] identified individual-level factors such as limited acclimatization, obesity, and existing health conditions that may heighten risk. Habibi et al. [40], Hokmabadi et al. [41], and Levi et al. [43] also identify that occupational heat exposure is directly linked with cognitive fatigue, irritability, and sleep disturbances as plausible contributors, mediated by dehydration increases the risks of occupational accidents. Despite heterogeneity, the reviews converge in suggesting that heat exposure can impair concentration and physical coordination, which may modestly increase accident probability. Evidence certainty for this relationship is low to moderate.

**Fig. 3:**
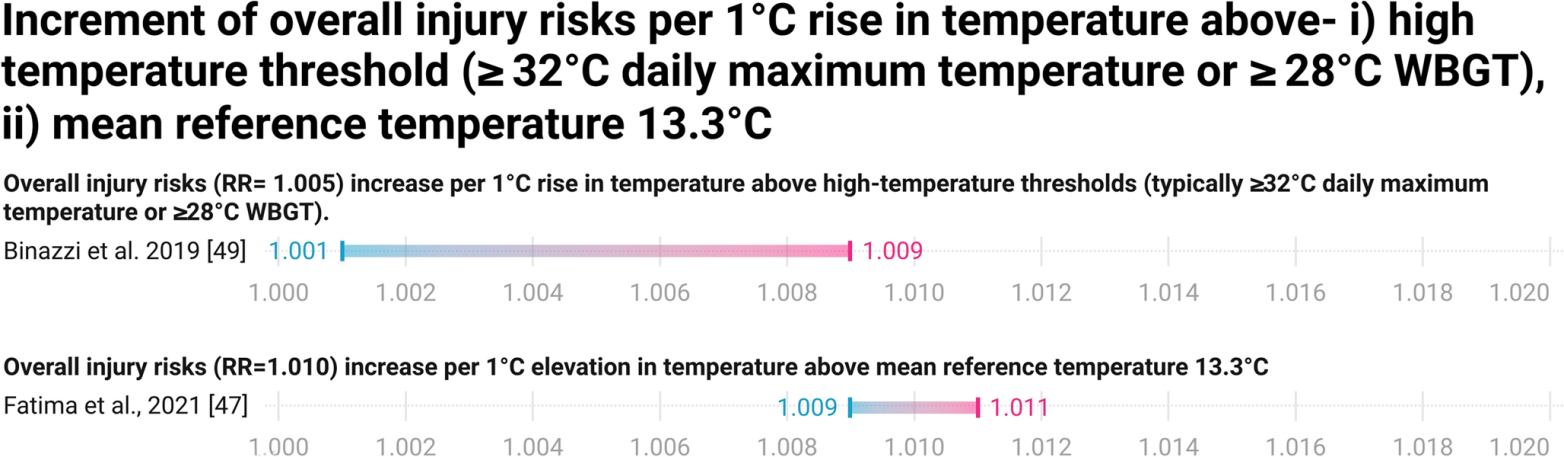
Increase in overall injury risks per 1 °C rise in temperature above thresholds

Quantification of overall injury risks is unambiguously presented in Fig. 3.

### Productivity Losses and Economic Implications

Five reviews examined productivity-related outcomes. Flouris et al. [39] and Han et al. [46] concluded that approximately 30%−60% workers suffer from productivity loss under high heat. Hokmabadi et al. [41] estimated that agricultural workers face up to a 63.9% productivity loss, while Han et al. [46] reported that construction workers experience up to a 62% productivity loss under prolonged high-temperature exposure. Levi et al. [43] and Zhao et al. [52] linked these individual effects to economic projections, suggesting that the potential labour productivity losses due to heat could yield global GDP losses of up to 2.6% by 2100 under high-emission scenarios. Overall, the evidence suggests that increased heat is related to decreased labour productivity.

### At-Risk Work Populations

Lee et al. [45] noted that rural workers and younger workers (15–35 years) were more susceptible to heat-related symptoms such as dehydration, headaches, cramps, and dizziness. Habibi et al. [50] found that women workers are more often fatigued and dehydrated compared to men, possibly due to differences in workload and clothing ensembles. Westwood et al. [48] identified other modifiers, such as advanced age, high body mass index, and failure to acclimatize. These findings should be interpreted cautiously because they are supported by a limited number of regional evidences and small subsamples. Nevertheless, reviews highlight the fact that biological, behavioural, and contextual factors, rather than a single demographic characteristic, determine predisposition to heat strain.

### Molecular Biomarkers of Heat Exposure

Habibi et al. [51] reviewed primary studies of biomarkers that may reflect oxidative stress from repeated heat exposure, including 8-hydroxy-2′-deoxyguanosine (8-OHdG), heat-shock protein 70 (HSP-70), and micronucleus frequency. The number of underlying studies was limited and heterogeneous; thus, the certainty of evidence was low. Currently, these markers are more exploratory indicators rather than established diagnostic markers. Validation in larger occupational cohorts is needed before routine application can be recommended.

### Strengths and Limitations

The strength of this umbrella review resides in its integrative scope, covering multiple occupational sectors and outcome domains (physiological, renal, cognitive, injury-related, molecular, and economic), and its reliance on review-level evidence, which minimizes individual study bias. By consolidating literature published between 2018 and 2024, it captures recent trends linking climate change and occupational health.

The use of the *AMSTAR* and *GRADE* frameworks enhanced methodological consistency and transparency. However, inherent limitations remain due to the reliance on observational evidence and the heterogeneity in exposure definitions and outcome measures. Moreover, the literature search and selection processes were carried out manually; while eligibility benchmarks were applied precisely, detailed exclusion records were not digitally archived, representing a limitation with regard to reproducibility. Therefore, all these factors taken together suggest that this umbrella review provides a robust but interpretative summary of the existing evidence, useful for guiding further research, refining exposure assessment, and informing targeted workplace heat-adaptation strategies.

## Conclusion

This umbrella review synthesizes consistent evidence linking occupational heat exposure to a range of adverse health outcomes, including renal impairment, cognitive decline, injuries, reproductive dysfunction, and DNA damage, as well as notable productivity losses. These effects are most pronounced in labour-intensive sectors such as agriculture, construction, and manufacturing, particularly in low- and middle-income countries where occupational protections are often inadequate.

While the overall certainty of evidence, as assessed by *GRADE*, ranged from low to moderate due to reliance on observational designs, variable exposure metrics, and heterogeneous outcome measures, the consistency across studies supports the validity of the findings.

Given the growing burden of heat-related health risks amid climate change, urgent, multi-level interventions are warranted. These include workplace adaptations, policy reforms, and gender- and migrant-inclusive heat safety standards. Future research should focus on standardized heat exposure assessment, longitudinal and interventional study designs, and targeted evaluations in high-risk occupational groups to guide effective and context-specific protective strategies.

## Supplementary Information

Below is the link to the electronic supplementary material.

Supplementary Material 1

Supplementary Material 2

## Data Availability

No datasets were generated or analysed during the current study.
