# Supplementary material for: An Umbrella Review of Systematic Reviews and Meta-Analyses on Occupational Heat Exposure, Health Risks, and Productivity Losses Globally
Source: Curr Environ Health Rep. 2026 Jan 7;13(1):2. doi: 10.1007/s40572-025-00520-8 (PMC12779733; doi:10.1007/s40572-025-00520-8)
Supplement: Supplementary file 1 — Supplementary Material 1 [file 40572_2025_520_MOESM1_ESM.docx]

Detailed of agreement between two individual reviewers (AN and SS) are enumerated in **Supplemental Material 2:**

|  | **SS: Include** | **SS: Exclude** | **Total** |
| --- | --- | --- | --- |
| **AN: Include** | a = 12 | b = 1 | 13 |
| **AN: Exclude** | c = 1 | d = 16 | 17 |
| **Total** | 13 | 17 | 30 |

Step by step calculation:

a) Observed Agreement (P_o_) = $\frac{(a+d)}{n}$ = $\frac{(12+16)}{30}$ = 0.933

Where, P_o_ is the proportion of cases where both reviewers agreed (Yes or No).

Here, a= 12 and d=16, n= 30 (total full text reviews assessed).

b) Expected Agreement (P_e_) = ($\frac{\left( a+b \right)(a+c)}{n^{2}}$) + ($\frac{(c+d)(b+d)}{n^{2}}$) = ($\frac{\left( 12+1 \right)(12+1)}{{30}^{2}}$) + ($\frac{(1+16)(1+16)}{{30}^{2}}$) = 0.5088 ≈ 0.509.

Where, P_e_ is the probability of agreement expected.

c) Cohen’s kappa κ = $\frac{\left( Po-Pe \right)}{1-Pe}$ = $\frac{\left( 0.933-0.509 \right)}{1-0.509}$ = 0.863.

Therefore, Cohen’s kappa (κ) indicates a strong level of agreement between the two independent reviewers (AN and SS) regarding the inclusion of the selected systematic reviews and meta-analyses.
